# Supplementary material for: A prevalence study in Guadalajara, Mexico, comparing tuberculin skin test and QuantiFERON-TB Gold In-Tube
Source: PLoS One. 2022 Mar 10;17(3):e0264982. doi: 10.1371/journal.pone.0264982 (PMC8912134; doi:10.1371/journal.pone.0264982)
Supplement: Attachment — Submitted filename: Response to Reviewers.docx [file pone.0264982.s002.docx]

**Distinguished Professor Lei Gao. Dec. 30. 2021**

**Academic Editor PLOS ONE**

**Distinguished Reviewers.**

**Present.**

**The co-authors of the manuscript originally titled:**

***Latent tuberculosis infection in Mexican pediatric AIDS patients.
A prevalence study in Guadalajara, Mexico, comparing Tuberculin Skin Test and QuantiFERON-TB Gold In-Tube.***

***(With the identification code: PONE-D-21-34415)***

**We would like to thank you for your kind comments made to the document. Likewise, below, we allow ourselves to answer your questions and comments.**

**Journal Requirements:**

1. When submitting your revision, we need you to address these additional requirements.

**ANSWER:** According to the observation; We have adhered to the style requirements of PLOS ONE.

2. Please amend your current ethics statement to address the following concerns……

**ANSWER:** According to the observation; The following change to ethical considerations can be identified: *“Ethical considerations. Because the investigation of LTBI (through TST and/or interferon gamma release assay, in HIV-patients, it’s a quality standard of care procedure, and fundamental to decide the use of chemoprophylaxis against active tuberculosis and the present study was based on the results of those tests, previously documented in the clinical records of the patients; it was sufficient to have the parents or guardian´s signature of the informed consent for general care of the minors, either as hospitalized or outpatient. Either way, this research was subjected to evaluation and authorized by the Committees of Research, Ethics and Biosafety both, of the Civil Hospital of Guadalajara and the Secretariat of Health of Jalisco Mexico.”*

3. Please review your reference list to ensure that it is complete and correct….

**ANSWER:** According to the observation; As can be seen in the revised manuscript, the references 7-10, 19, 23, and 32 were corrected; Likewise, references 34, 35 and 36 were incorporated, to support some concepts that address observations made by the editor and by the reviewers.

**Additional Editor Comments**

Please discuss the limitations of the study.

**ANSWER:** According to your valuable observation, in the new revised manuscript we have added this paragraph: “*Our study has some limitations. Although QFT was notoriously more sensitive than TST, the limited sample size (48 patients) probably influenced the finding of no statistical significance of the differences. Likewise, in a future study it would be interesting to compare different IGRAs such as QFT-GIT and Enzyme Linked Immune absorbent spot [ELISPOT] in the diagnosis of LTBI and active TB in children with HIV infection. It should be considered that our study was carried out in only one of the largest reference hospitals in Mexico and that could not fully reflect the situation in the country. A multicenter study in Mexico including several regions would be interesting. Finally, as with other studies, it is difficult to establish the sensitivity and specificity of these tests in the diagnosis of LTBI, since there is no comparative gold standard, as if there is one for active tuberculosis.*”

Please discuss why no TST+/QFT- was observed given most of the studied children were BCG vaccinated.

**ANSWER:** It is an excellent and valuable comment; We have added this paragraph which we think may explain the observation: “*this can be explained by the fact that 81% of the children were over 6 years of age and all those vaccinated, received BCG in the first three months of life, which is consistent with the consideration that more than 5 years after vaccination a positive TST could be indicative of LTBI and less probably positive-false due to the vaccine. In fact, in a recent study carried out in India whose objective was to determine the concordance between QFT-GIT and TST in 33 children (without HIV) vaccinated with Bacillus Calmette-Guerin, a false positive rate of TST of 83.3% and 33.3% among children under and over 4 years of age was respectively estimated, which suggests that more years after vaccination, the lower the likelihood of false positive TST [34]”*.

**Reviewer #1, Professor Layth Al-Salihi:**

 This study showed a scientific flow in its context, using suitable epidemiological model and correct statistical testing. It showed that IGRA test can label positive cases more than TST but a constrain is that cannot know which is right as there is no gold standard test is available yet.

**ANSWER:** Fully agree with your observation; in the new revised manuscript we have decided to add the following paragraph: *“Identifying the sensitivity and specificity of TST and IGRAs in the diagnosis of LTBI represents a great difficulty, because in that setting there is not a comparative "gold standard test"; on the other hand, it is easier to define them for active tuberculosis, by having more specific elements and benchmarking objectives such as MTB cultures or molecular tests such as the MTB-Xpert MTB-RIF assay, or the combination of a TB-compatible clinical picture with findings in imaging studies, positive Ziehl–Neelsen staining smears and/or histological studies.”*

**Reviewer #2 Professor Shibu Balakrishnan:**

The study is relevant and well conducted...…. Two suggestions are noted for the consideration of authors.

The inclusion and exclusion criteria adopted by the authors may be mentioned.

**ANSWER:** Fully agree with your observation; in the new revised manuscript we have decided to add the following paragraph*: “Exclusion criteria. Patients with active or past TB disease were excluded from the study*”.

AIDS patient, HIV patient are some of the nomenclatures used interchangeably by the authors…...

**ANSWER:** Totally according to your observation, in the new revised manuscript we have decided to use the terms "HIV-infected", "HIV-infection", "HIV-patients" instead of AIDS or AIDS patients.

**Reviewer #3:**

It's an interesting work. It could be more interesting if the analysis used not only QuantiFeron test but also the ELISPOT test and comparing both results; data comparing results with both technics are very important as they show that ELUSPOT is better the QUANTIFeron for the diagnosis of LTBI.

**ANSWER:** According to your valuable observation, in the new revised manuscript we have added two paragraphs: “*Comparative evaluation of IGRAs in the diagnosis of LTBI in children with HIV infection is certainly an area of interest. Some studies have shown high levels of agreement (93%, κ = 0.83) between QFT-IT and and an enzyme-linked immunospot assay (ELISPOT) in children at risk of LTBI or active tuberculosis [35]. A meta-analysis to assess different IGRAs (QFT-G, QFT-GIT and Enzyme Linked Immune absorbent spot [ELISPOT]) and TST found that the sensitivity of the three tests in children with active TB were similar. Pooled specificity was 100% for QFT-based tests and 90% for ELISPOT, but was much lower for TST (56% overall and 49% in children with BCG vaccination). [36]”* and *“Likewise, in a future study it would be interesting to compare different IGRAs such as QFT-GIT and Enzyme Linked Immune absorbent spot [ELISPOT] in the diagnosis of LTBI and active TB in children with HIV infection.”*

As data are of a Guadalajara in Mexico, do you know a similar data in another location of Mexico? May be interesting if it could compare similar data in the country.

**ANSWER:** According to your observation, in the new revised manuscript we have added this paragraph: *“Our study identified a prevalence of LTBI in HIV-infected children (under 16 years of age) of 16.6% based on QFT, in one of the largest teaching hospitals, located in the central-western region of the country and that was lower than that reported in a study in children with HIV infection living in the border region between Tijuana, Mexico and San Diego California, United States using TST and QTF-GIT, where a prevalence of LTBI of 20.3% was identified [11]. This could be a reflection of the higher incidence of tuberculosis in the U.S.-Mexico border region, which accounts for 30% of the total TB cases recorded in both countries [33].”*

**Respectfully.**

**Héctor Raúl Pérez Gómez PhD. Corresponding autor.**
